# Influence of Ion Size on Structure and Redox Chemistry in Na‐Rich and Li‐Rich Disordered Rocksalt Battery Cathodes

**DOI:** 10.1002/adma.202419878

**Published:** 2025-05-30

**Authors:** Nicole C. Mitchell, Oliver O. Thomas, Benjamin G. Meyer, Mirian Garcia‐Fernandez, Ke‐Jin Zhou, Patrick S. Grant, Peter G. Bruce, Richard Heap, Ruth Sayers, Robert A. House

**Affiliations:** ^1^ Department of Materials University of Oxford Parks Road Oxford OX1 3PH UK; ^2^ The Faraday Institution Didcot OX11 0RA UK; ^3^ Diamond Light Source Harwell Campus Didcot OX11 0DE UK; ^4^ Faradion Ltd. The Innovation Centre Sheffield S1 4DP UK

**Keywords:** disordered rocksalts, li‐ion cathodes, li‐rich, na‐ion cathodes, na‐rich, oxyfluorides

## Abstract

Li‐rich disordered rocksalts are promising next‐generation cathode materials for Li‐ion batteries. Recent reports have shown it is also possible to obtain Na‐rich disordered rocksalts, however, it is currently poorly understood how the knowledge of the structural and redox chemistry translates from the Li‐rich to the Na‐rich analogs. Here, the properties of Li_2_MnO_2_F and Na_2_MnO_2_F are compared, which have different ion sizes (Li^+^ = 0.76 vs Na^+^ = 1.02 Å) but the same disordered rocksalt structure and stoichiometry. It is found that Na_2_MnO_2_F exhibits lower voltage Mn‐ and O‐redox couples, opening access to a wider compositional range within the same voltage limits. Furthermore, the intercalation mechanism switches from predominantly single‐phase solid solution behavior in Li_2_MnO_2_F to a two‐phase transition in Na_2_MnO_2_F, accompanied by a greater decrease in the average Mn─O/F bond length. Li_2_MnO_2_F retains its long‐range disordered rocksalt structure throughout the first cycle. In contrast, Na_2_MnO_2_F becomes completely amorphous during charge and develops a local structure characteristic of a post‐spinel. This amorphization is partially reversible on discharge. The results show how the ion intercalation behavior of disordered rocksalts differs dramatically when changing from Li‐ to Na‐ions and offers routes to control the electrochemical properties of these high‐energy‐density cathodes.

## Graphical abstract

The larger ion size of Na^+^ versus with Li^+^ has a pronounced impact on the intercalation chemistry of disordered rocksalt cathode materials. On charge, the disordered rocksalt structure is retained in Li_2_MnO_2_F but it is lost in Na_2_MnO_2_F resulting in extensive amorphization. The larger ion size also lowers the average voltage enabling higher capacities between equivalent voltage limits.

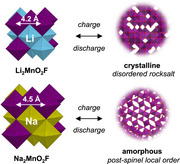

## 1 Introduction

Increasing the energy density of rechargeable batteries would significantly accelerate the transition to Net Zero by enabling electric vehicles with longer driving ranges.^[^ ^1^ ^]^ A key limitation of current battery technology is the cathode material. The highest energy density commercially available cathodes for Li‐ and Na‐ion batteries are based on stoichiometric layered transition metal (TM) oxides, such as LiNi_0.8_Mn_0.1_Co_0.1_O_2_ and NaNi_1/3_Fe_1/3_Mn_1/3_O_2_.^[^ ^2^, ^3^ ^]^ Alkali‐rich compounds (i.e., where Li:TM > 1 or Na:TM > 1) offer a route to further increase the energy density, however, Li‐rich layered oxides suffer from pronounced structural change and there are very few known examples of Na‐rich layered oxides due to the large mismatch in ion size between TMs and Na^+^.^[^ ^4^, ^5^ ^]^ Alkali‐rich disordered rocksalts are a promising class of cathode materials which have been shown to exhibit remarkable structural integrity over wide composition ranges.^[^ ^6^, ^7^, ^8^, ^9^, ^10^, ^11^, ^12^ ^]^ Li‐rich disordered rocksalt cathodes, such as Li_1.3_Nb_0.3_Mn_0.4_O_2_ and Li_2_MnO_2_F, can retain their structure even when >1 Li is removed.^[^ ^13^, ^14^ ^]^

The effect of Li/Na ion size on the voltage profile of layered structures has been well‐documented. For instance, the interlayer spacing in NaCoO_2_ is larger than that of LiCoO_2_, and its average voltage is lower. This has been attributed to the larger ionic radius of Na^+^ compared to Li^+^, which increases the interlayer spacing, reducing the electrostatic attraction between layers, and consequently lowering the energy required to remove Na^+^ ions during charging.^[^ ^15^, ^16^, ^17^ ^]^ Differences in ion size between Li^+^ and Na^+^ also play an important role in determining the structural transitions cathodes undergo during charge. Sodium‐layered oxides often exhibit multiple stacking sequence transitions between P‐ and O‐type structures due to the large ion size of Na^+^.^[^ ^15^, ^18^, ^19^ ^]^ Recent work has shown that ion size is central to predicting the phase stability of P‐ versus O‐type structures.^[^ ^20^ ^]^

Despite a well‐established understanding on the role of ion size in layered materials, it is not clear how this translates to disordered rocksalt materials which do not have ordered layers. In this study, the influence of ion size on the structural evolution and redox chemistry in isostructural disordered rocksalts Li_2_MnO_2_F and Na_2_MnO_2_F is investigated. Since Na^+^ is larger than Li^+^, Na_2_MnO_2_F exhibits a larger cubic lattice parameter than Li_2_MnO_2_F. Accompanying this difference, we find that the average charging voltage of Na_2_MnO_2_F is also lower than Li_2_MnO_2_F, even after accommodating for the 0.3 V difference in potential of the Na and Li counter electrode. Consequently, a significantly greater degree of ion deintercalation can be achieved in Na_2_MnO_2_F than Li_2_MnO_2_F over equivalent voltage windows (2 Na vs 1.25 Li) and more trapped O_2_ can be formed in the lattice.

We also find pronounced differences in phase evolution behavior during charge and discharge. While Li_2_MnO_2_F undergoes uniform lattice contraction and expansion, Na_2_MnO_2_F loses its long‐range disordered rocksalt structure and becomes amorphous via a two‐phase conversion mechanism. The local structure of Na_2_MnO_2_F also undergoes pronounced changes from disordered rocksalt to a dense, post‐spinel configuration which is only partially reversed on discharge. This transition is accompanied by a 10.3% decrease in average Mn─O/F bond length as the structure relaxes from the removal of oversized Na^+^ ions. For Li_2_MnO_2_F, the decrease, 2.4%, is smaller as the Mn─O/F bond lengths are much closer to the values they converge toward in MnO_2_F. Interestingly, both of these charging mechanisms are able to support class‐leading energy densities of 900 Wh kg^−1^ in Li_2_MnO_2_F and 610 Wh kg^−1^ in Na_2_MnO_2_F. Mastering the intercalation chemistry of disordered rocksalts is critical to further improving the performance of these next‐generation cathode materials.

## 2 Main Text

**Figure 1:**
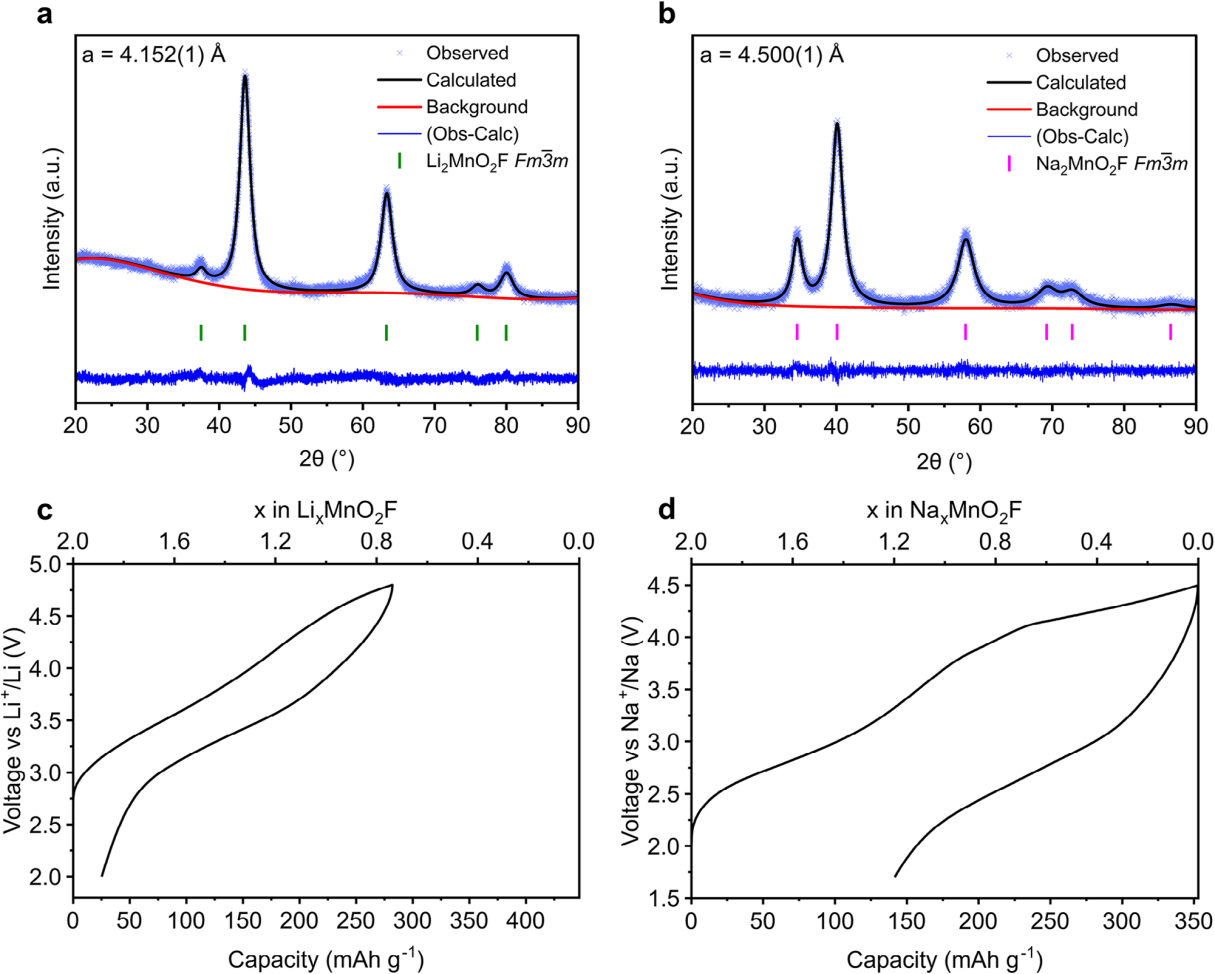
Structure and electrochemistry. Rietveld refinements of powder X‐ray diffraction data for a) Li_2_MnO_2_F and b) Na_2_MnO_2_F disordered rocksalts, with lattice parameters 4.15 and 4.50 Å respectively. Electrochemical load curves for the first cycle of c) Li_2_MnO_2_F and d) Na_2_MnO_2_F over equivalent voltage windows of 2–4.8 V versus Li^+^/Li and 1.7–4.5 V versus Na^+^/Na.

Li_2_MnO_2_F and Na_2_MnO_2_F were prepared by mechanochemical ball‐milling as detailed in Methods. Li_2_MnO_2_F was prepared by the same route as reported previously, **Figures** **1**a, S1a (Supporting Information).^[^ ^14^, ^21^ ^]^ Na_2_MnO_2_F was first targeted by Moghadam et al.,^[^ ^22^ ^]^ using a mixture of Na_2_O_2_ and Na_2_O precursors as the Na source, however, given the high impurity level of Na_2_O_2_ (≈20%) present in commercial Na_2_O, we developed an alternative synthesis method using NaMnO_2_ and NaF as precursors. The powder X‐ray diffraction (PXRD) data in Figure 1b, show that single‐phase Na_2_MnO_2_F can be obtained by this route with a uniform elemental distribution across particles, shown by energy dispersive X‐ray spectroscopy (EDX) analysis, Figure S1b (Supporting Information). The refined unit cell lattice parameter for Na_2_MnO_2_F, 4.50 Å, is slightly larger than previous reports^[^ ^22^ ^]^ and substantially larger than that of Li_2_MnO_2_F, 4.15 Å. This result is consistent with the larger ionic radius of Na^+^ (1.02 Å) than Li^+^ (0.76 Å). The average crystallite size estimated from the peak width using Scherrer analysis is comparable between both samples (4.6 nm compared to 5.6 nm), and the particle sizes and morphologies are similar from scanning electron microscopy (SEM), Figure S2a,b (Supporting Information). The oxidation state of Mn was verified as +3 in both cases via iodometric titration, Table S2 (Supporting Information).

The first cycle load curves for Li_2_MnO_2_F and Na_2_MnO_2_F are shown in Figure 1c,d. The cathodes were cycled over equivalent voltage windows (2–4.8 and 1.7–4.5 V) accommodating for the 0.3 V difference in reference potential of the Li and Na counter electrodes. Interestingly, the full Na content of the Na_2_MnO_2_F material can be extracted, while in contrast only a little over half of the Li is accessible in Li_2_MnO_2_F. This represents one of the widest compositional windows ever reported for a Na‐ion cathode material. On discharge, 1.2 Na can be reversibly re‐intercalated which is comparable to 1.2 Li for the Li analog, corresponding to capacities of 210 and 250 mAh g^−1^ respectively.

The average voltage of the extraction of 1 Na is 3 V, compared with 3.7 V for 1 Li, Figure S3 (Supporting Information). Even after the 0.3 V adjustment in potential, the average voltage of the Na extraction process is 0.4 V lower than Li. This indicates that the oxidation potential of the Mn^3+/4+^ redox couple is lower in Na_2_MnO_2_F than Li_2_MnO_2_F. The reason for this could be due to differences in the strength of bonding between Li─F and Na─F.^[^ ^23^ ^]^ However, we find a similar difference in average voltage on charge of 0.35 V between NaMnO_2_ and LiMnO_2_, indicating this is largely independent of the identity of the anion, Figure S4 (Supporting Information). Instead, given the only main structural difference between the compounds is the lattice parameter, we attribute the lower voltage of Mn‐redox in Na_2_MnO_2_F to the longer average bond lengths of Mn─O/F (2.25 Å compared with 2.08 Å in Li_2_MnO_2_F), caused by lattice expansion from the large Na^+^ ion. Elongating the metal‐ligand bond lengths reduces the extent of orbital overlap raising the energy of the Mn3d‐O2p/F2p bonding states and reducing the voltage.

The same effect also appears to influence the oxidation potential of O^2−^. The voltage upon charge at which 1 Na has been extracted (3.8 V), which is nominally the point at which the redox process switches from Mn oxidation to O oxidation, is 0.7 V lower than the voltage at which 1 Li has been extracted. Subsequently, a much longer region of O‐redox charge capacity is observed beyond Mn^4+^ for Na_2_MnO_2_F than Li_2_MnO_2_F. This modulation of the redox potentials is likely to be responsible for the significantly higher amount of Na than Li that is accessible in these disordered rocksalt cathodes.

**Figure 2:**
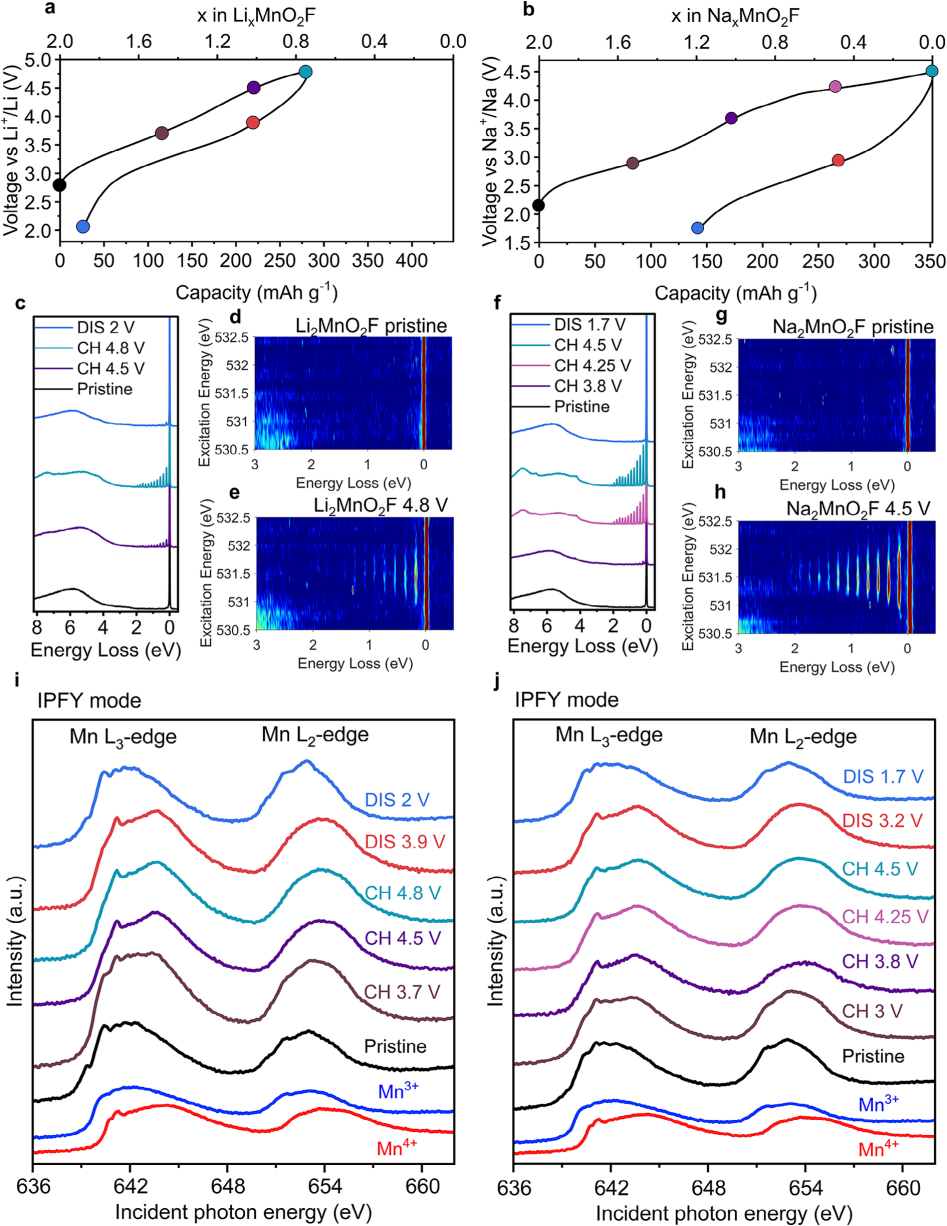
O K‐edge and Mn L‐edge spectroscopy. States of charge for ex situ analysis in a) Li_2_MnO_2_F and b) Na_2_MnO_2_F. c,f) O K‐edge RIXS data collected at an excitation energy of 531.5 eV. d,e,g,h) O K‐edge RIXS maps between excitation energies 530.5–532.5 eV showing the vibrational peaks arising from molecular O_2_ trapped in the bulk. Mn L‐edge XAS in inverse partial fluorescence yield (iPFY) mode for i) Li_2_MnO_2_F and j) Na_2_MnO_2_F. Mn oxidation from +3 to +4 is followed by O^2−^ oxidation to O_2_ trapped in the bulk of cathode particles. Mn^3+^ reference Mn_2_O_3_ and Mn^4+^ reference MnO_2_.

To investigate the charge compensation processes accompanying ion (de)intercalation in the Na and Li disordered rocksalt analogs, Mn L‐edge soft X‐ray absorption spectroscopy (XAS) in bulk sensitive inverse partial fluorescence yield mode (iPFY),^[^ ^24^ ^]^ and O K‐edge resonant inelastic X‐ray scattering (RIXS) were performed, **Figure** **2**. The Mn L‐edge XAS data show Mn oxidation from +3 to +4 occurs predominantly over the lower voltage region of the load curve corresponding to the extraction of the first mole of Li and Na, Figure 2i,j. Upon further charging there is no significant change in the edge position indicating the Mn remains in the +4 oxidation state.

The O K‐edge RIXS data over the first charge process show that appearance of a series of sharp peaks between 0–2 eV energy loss, characteristic of molecular O_2_ trapped within the structure as reported previously in O‐redox battery cathodes.^[^ ^25^, ^26^, ^27^, ^28^, ^29^, ^30^ ^]^ This trapped O_2_ formed as the product of O^2−^ oxidation begins to appear predominantly when charging beyond the first mole of Li and Na, Figure 2a–h. A significantly higher signal intensity of O_2_ is observed for fully charged Na_2_MnO_2_F than Li_2_MnO_2_F reflecting the higher O‐redox capacity observed in the former, Figure 2c–h. On discharge, little evidence remains of the trapped O_2_ formed electrochemically during charge, indicating reduction back to O^2−^. The Mn L‐edge XAS line‐shapes also return to those of the pristine materials, reflecting a reduction to Mn^3+^. These redox changes accord with previous observations for Li_2_MnO_2_F,^[^ ^14^, ^21^, ^31^ ^]^ with some evidence of overlap between the Mn and O redox couples on charge as shown by the presence of a small amount of O_2_ in each material at the charge point equivalent to extraction of 1 Li and 1 Na. Interestingly, the Mn and O redox couples appear to be less overlapped in Na_2_MnO_2_F than Li_2_MnO_2_F. Comparing the RIXS spectra at the nominal point of cross‐over between the redox couples, i.e., 1 mole of Na or Li extracted, there appears to be less trapped O_2_ formed in the Na analog, Figure S5 (Supporting Information). This could be another consequence of the elongated Mn─O/F bonds due to the large size of Na ions. The reduced orbital overlap between Mn and O/F will also separate the Mn3d states from the O2p and F2p states in energy, making the bond more ionic and promoting the separation of the Mn and O‐redox couples.

**Figure 3:**
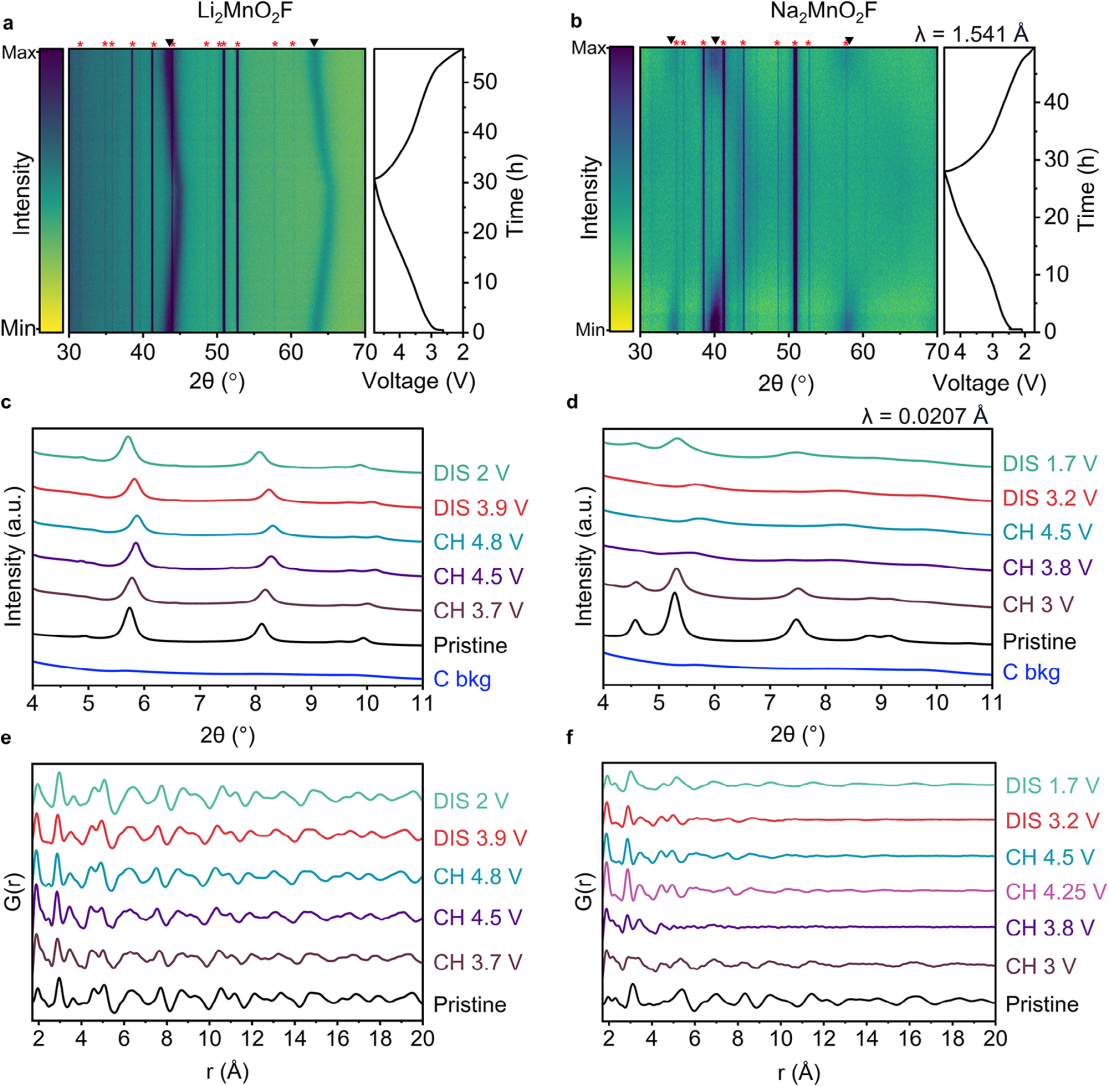
Phase evolution over first cycle. Operando powder X‐ray diffraction data for a) Li_2_MnO_2_F and b) Na_2_MnO_2_F using lab‐source Cu Kα_1,2_ X‐ray radiation and current density of 20 mA g^−1^. Peaks marked with an asterisk (^*^) arise from the operando cell components and the peaks marked with a triangle (^q^) are from the disordered rocksalts. c,d) ex situ synchrotron powder X‐ray diffraction data with wavelength λ = 0.0207365 nm. e) Li_2_MnO_2_F and f) Na_2_MnO_2_F ex situ PDF analysis of X‐ray total scattering data. The long‐range cubic rocksalt structure is retained for Li_2_MnO_2_F, but lost for Na_2_MnO_2_F during first charge. Li_2_MnO_2_F exhibits single‐phase evolution over the first cycle, but Na_2_MnO_2_F charges via a two‐phase mechanism. The charged phase for Na_2_MnO_2_F is amorphous, but can be partially re‐crystallised into a disordered rocksalt structure upon Na^+^ re‐intercalation on discharge.

To understand the structural changes accompanying the charge and discharge, operando powder X‐ray diffraction was performed on each electrode. The results in **Figure** **3**a,b, show continuous contraction and expansion of the cubic rocksalt structure for Li_2_MnO_2_F with charge and discharge respectively, characteristic of solid solution behavior in accord with previous reports.^[^ ^14^, ^21^ ^]^ The average Mn─O and Mn─F bond lengths change from 2.08 to 2.02 Å equivalent to a 7.5% reduction in unit cell volume. In contrast, for Na_2_MnO_2_F, rather than shifting continuously in scattering angle, the rocksalt diffraction peaks diminish in intensity and disappear upon desodiation giving rise to two very broad, low‐intensity peaks at ≈2θ = 5.7 and 8.3 °, Figure 3c,d, indicative of a two‐phase transition.

**Figure 4:**
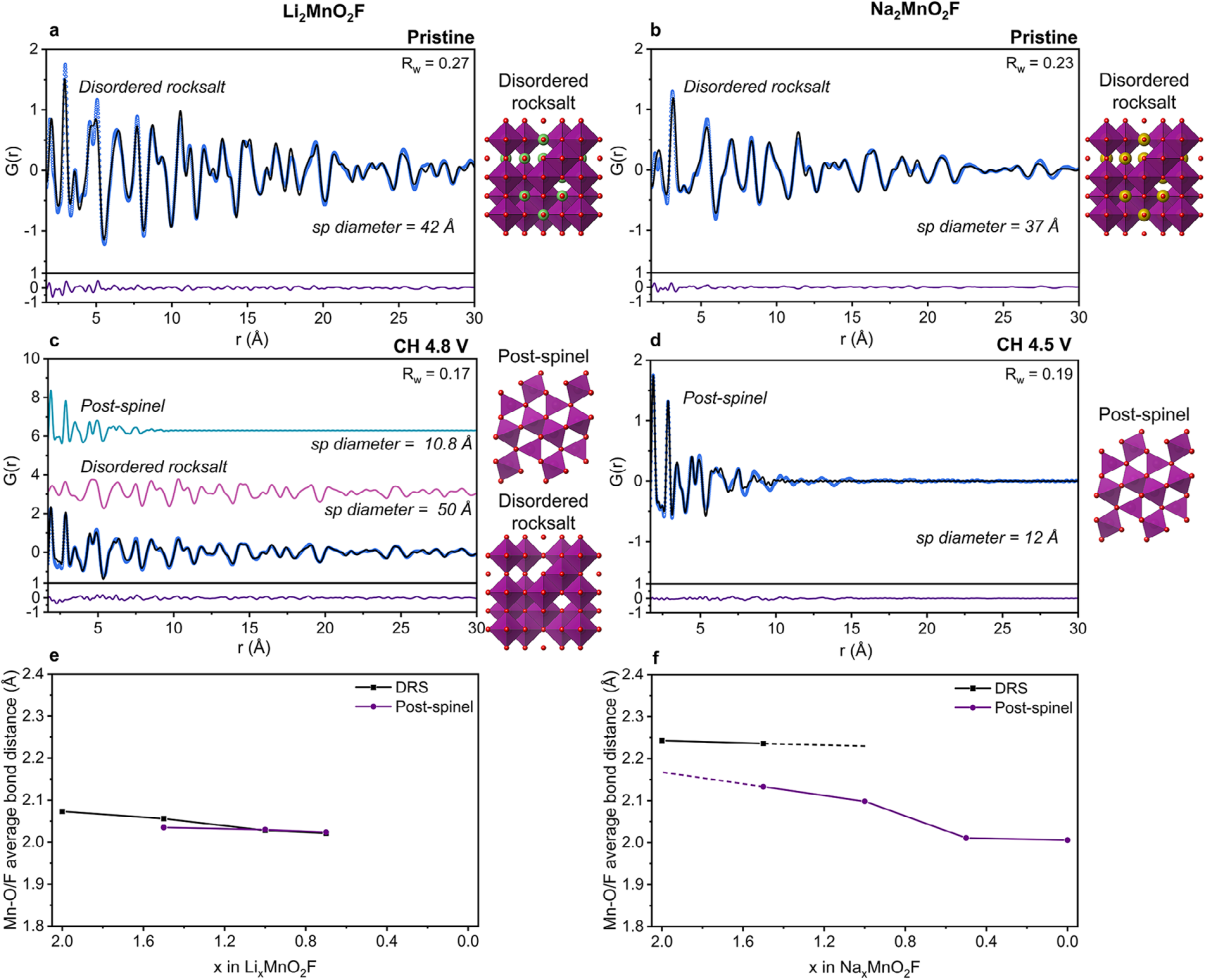
Local structure. Fitted X‐ray PDFs for a,c) Li_2_MnO_2_F and b,d) Na_2_MnO_2_F in the pristine and charged states. Color coding for atoms: green = Li, purple = Mn, red = O, grey = F, yellow = Na, white = vacancy. Color coding for figures: blue circles = observed data, solid lines = calculated PDFs. e,f), Average Mn─O/F bond lengths at different states of charge for Li_2_MnO_2_F and Na_2_MnO_2_F. The Li analog exhibits a gradual contraction of the rocksalt lattice during delithiation with a 2.4% decrease in average Mn─O/F bond length. The shortening of the average Mn─O/F bond length is much more severe in Na_2_MnO_2_F, reducing by 10.3% over the full range of Na content. This pronounced bond contraction is accompanied by a loss of the disordered rocksalt crystal structure. The local structure of the charged Na1 and Na0 phases is similar and is well‐described by arranging Mn and O/F in a post‐spinel structure (Pbcm space group).

Given the limited structural data available from long‐range Bragg scattering, ex situ pair distribution function (PDF) analysis of the X‐ray total scattering was carried out to study the changes in local structure in more detail. The PDFs for pristine Na_2_MnO_2_F and Li_2_MnO_2_F can be fitted well using a disordered rocksalt Fm‐3m structural model, **Figure** **4**a,b, with average Mn‐O/F bond lengths (2.24 Å and 2.07 Å) matching those from Rietveld refinement (2.25 Å and 2.08 Å). The local structure over the region 1.7–10 Å can also be fitted well using a disordered rocksalt model indicating any short‐range order present is not as extensive as seen in other compounds,^[^ ^32^, ^33^, ^34^ ^]^ supplementary Figure S13 (Supporting Information).

Upon charging, the PDFs for Li₂MnO₂F (Figures 3, 4) remain consistent with the disordered rocksalt structure. The PXRD data (Figure 3c) show that the long‐range correlation associated with the disordered rocksalt structure is retained throughout cycling.

In contrast, for Na_2_MnO_2_F there is a significant loss of PDF intensity at high r, corresponding to the loss of long‐range disordered rocksalt structure. In its place, a new local structure emerges (Figures 3, 4). The size of the ordered domains reduces from ≈40 to 10 Å, which accords with the loss of Bragg scattering intensity and indicates the material has become amorphous. While the partial loss of crystallinity has been observed previously in disordered rocksalts such as Li_8/7_Ti_2/7_V_4/7_O_2_,^[^ ^35^ ^]^ the near‐complete amorphization we observe for Na_2_MnO_2_F is unusual among this class of material. Interestingly, these pronounced changes to the average and local structure in Na_2_MnO_2_F occur early on in the charging process, before the onset of oxygen redox, implying that amorphization is not triggered by the formation of trapped O_2_. The amorphous nature of Na_2_MnO_2_F in the charged state was previously identified by Moghadam et al.^[^ ^22^ ^]^ using high‐angle annular dark‐field scanning transmission electron microscopy, however, the local structure of the material remains poorly understood and so too the nature of the crystalline‐amorphous transition.

Upon discharging, the disordered rocksalt structure of Li_2_MnO_2_F remains intact, with a gradual increase in lattice parameter as Li is re‐intercalated, Figure 3a,c. In the case of Na_2_MnO_2_F, the amorphous charged phase partially recrystallizes into a disordered rocksalt as evidenced by the recovery of the Bragg peaks in the PXRD pattern, Figure 3b,d. Fittings of the PDF data in the fully discharged state at 1.7 V, show that some of the amorphous material remains, Figure S11 (Supporting Information).

The local structure of the fully charged phase for Na_2_MnO_2_F was examined against a wide variety of known manganese oxide and fluoride compounds. The best fit was obtained using a Mn_3_O_4_ post‐spinel compound based on the orthorhombic Pbcm structure type.^[^ ^36^ ^]^ A comparison of the fittings for disordered rocksalt, spinel, and post‐spinel structures is included in Figure S12 (Supporting Information). The occupancies of the Mn sites were freely refined yielding a Mn: anion ratio of 1:3. This is consistent with the global composition at the top of charge, MnO_2_F. Given that the X‐ray scattering lengths of O and F are very similar in our X‐ray data, it is not possible to distinguish them in our refinement.

Closer inspection of the local structure on charge reveals that the average Mn─O/F distance has decreased substantially from 2.24 Å in the pristine material to 2.01 Å, Figure 4f. This is a significantly more pronounced change than occurs in Li_2_MnO_2_F (2.07 to 2.02 Å), Figure 4e. Two‐phase refinements performed on the PDFs at intermediate states of charge, show that the disordered rocksalt and post‐spinel phases co‐exist on charge with significantly different average bond distances, consistent with the operando powder X‐ray diffraction data. This reinforces the interpretation of a two‐phase charging mechanism for Na_2_MnO_2_F in contrast with the single‐phase solid solution observed for Li_2_MnO_2_F. For Li_2_MnO_2_F, the disordered rocksalt phase remains as the dominant phase throughout the charge. The emergence of some local regions of post‐spinel can be seen, in Figure 4c, with very similar average bond distances to the rocksalt, Figure 4e, indicating they likely exist as domains within the disordered rocksalt structure.

## 3 Discussion

The direct comparison between Li_2_MnO_2_F and Na_2_MnO_2_F in this study allows us to draw out the unique intercalation chemistry of disordered rocksalt cathode materials imposed by the difference in ion size between Li and Na. As observed by PDF, the larger ion size results in a bigger change in the Mn─O/F bonds during the charge for Na_2_MnO_2_F than Li_2_MnO_2_F. In order to minimize the large local strain that such a difference in bond length will induce, phase segregation into a sodiated and desodiated phase is preferable to a uniform, solid‐solution depletion of sodium. The removal of Na ions from Na_2_MnO_2_F also triggers the collapse of the long‐range disordered rocksalt structure to form an amorphous charged phase. Our PDF fittings show the local structure of this phase is best described as a post‐spinel, which is a denser, more compact structure than the related spinel phases.

This two‐phase, crystalline‐amorphous charging mechanism is in stark contrast to the single‐phase charging mechanism observed in Li_2_MnO_2_F and most other Li‐rich disordered rocksalt cathodes and bears closer resemblance to the behavior of conversion cathodes.^[^ ^37^ ^]^ Metal oxide and fluoride conversion cathodes also typically undergo complex phase transitions involving several distinct phases with differing degrees of crystallinity.^[^ ^38^, ^39^, ^40^ ^]^ The electrochemical response of Na_2_MnO_2_F also shares many of the hallmarks of conversion cathodes such as voltage hysteresis and large specific capacities.

The role of ion size is well‐understood in ordered, layered compounds, with larger Na‐containing materials exhibiting stacking sequence transitions during desodiation and more dramatic changes in interlayer spacing which can be detrimental for cycling performance. In these systems, pillaring with alternative cations, such as K, Ca, and Sr, is a commonly employed strategy to stabilize the structure and mitigate against these changes.^[^ ^41^, ^42^, ^43^ ^]^ Our results show that ion size is also an important factor in determining the structural behavior of disordered rocksalts, and would merit further investigation as a lever for controlling performance.

The large ion size of Na may also explain why it has not yet proved possible to synthesize Na‐rich disordered rocksalts directly via solid‐state methods. Most Li‐based rocksalts use either Li_2_TiO_3_ or Li_3_NbO_4_ as end‐member phases to promote cation disorder as they crystallize as disordered rocksalts. However, neither Na_2_TiO_3_ or Na_3_NbO_4_ are found to crystallize as disordered rocksalts despite the presence of d^0^ transition metal ions. The large mismatch in size between Na (1.02 Å) and Ti or Nb (0.61 and 0.68 Å, respectively) is likely to promote the formation of ordered phases to minimize local strain.

Ion size may also influence the ion diffusion mechanism in disordered rocksalt cathodes. In Li‐ion disordered rocksalt materials, Ceder and co‐workers proposed that the size of the tetrahedral interstitial sites is critical to allowing Li diffusion between adjacent octahedral sites.^[^ ^6^ ^]^ In our oxyfluoride materials, it appears that the average size of the tetrahedral interstitial sites scales in the same way as the ion size, Table S13 (Supporting Information). Similar mechanisms of diffusion for Li and Na would therefore seem plausible, although further work would be needed to investigate this in detail.

**Figure 5:**
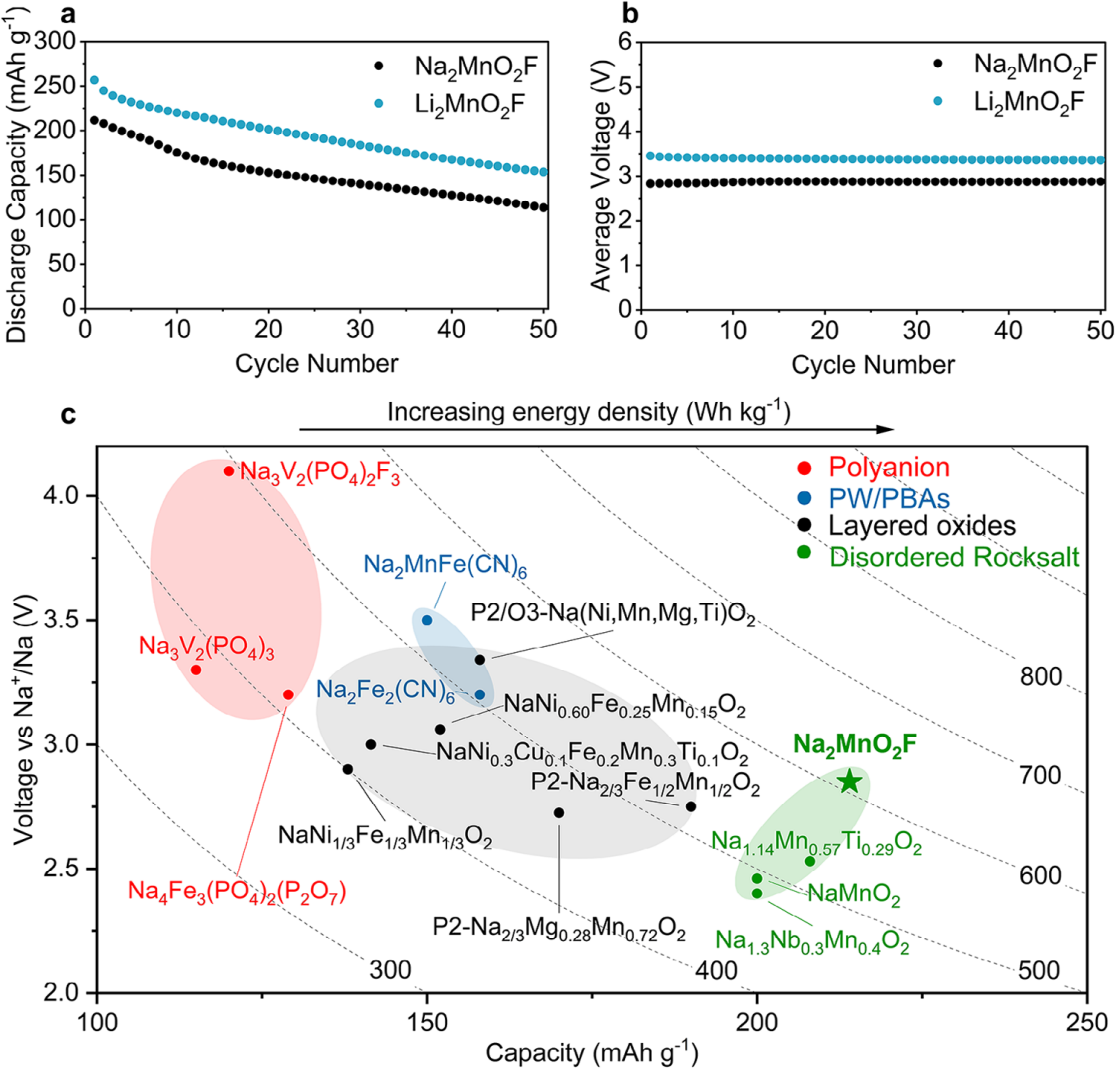
Electrochemical performance. a) Discharge capacity and b) average discharge voltage retention over the first 50 cycles at 10 mA g^−1^ in Na and Li half‐cells, for Na_2_MnO_2_F and Li_2_MnO_2_F respectively. c) Average voltage versus capacity plot for polyanion, Prussian White analogs and layered oxide cathode classes. Na_2_MnO_2_F exhibits one of the largest energy densities reported among Na‐ion cathodes.^[^ ^30^, ^47^, ^48^, ^49^, ^50^, ^51^, ^52^, ^53^, ^54^, ^55^, ^56^, ^57^, ^58^, ^59^ ^]^

Na_2_MnO_2_F exhibits one of the highest energy densities ever reported among Na‐ion cathodes (>600 Wh kg^−1^), **Figure** **5**c, and similar capacity and voltage retention to the lithium analog, Figure 5a,b, Figure S14 (Supporting Information). Na‐rich disordered rocksalts could therefore offer a promising route to achieving higher energy density providing the origin of the capacity fade can be suppressed. It has recently been shown that increasing electronic connectivity within the composite electrode can significantly improve capacity retention in Li‐rich manganese oxyfluorides.^[^ ^44^, ^45^ ^]^ This mitigates against the insulating cathode electrolyte interphase which forms on the surface of the cathode particles over cycling.^[^ ^45^ ^]^ We expect that similar strategies such as carbon coatings and use of carbon nanofibres rather than powders can be employed for the Na‐rich analogs to improve their cycling performance. O‐redox has also been shown to exacerbate performance degradation in disordered rocksalts. Lowering the upper‐cutoff voltage below the onset of O─O dimerisation can avoid these issues and lead to significant improvements in capacity retention,^[^ ^46^ ^]^ Figure S14 (Supporting Information).

## 4 Conclusion

Li‐rich disordered rocksalt cathodes offer some of the highest known energy densities for Li‐ion batteries. However, it is not yet understood how the electrochemical properties of well‐studied Li‐rich cathodes translate to the Na‐rich analogs. Here, we compare the structural and redox properties of Li_2_MnO_2_F and Na_2_MnO_2_F battery cathodes. We show that the larger ion size of Na compared with Li results in lower average voltages for Mn^3+/4+^ and O‐redox. Consequently, higher capacities can be achieved within equivalent voltage limits and all of the Na can be extracted from Na_2_MnO_2_F below 4.5 V. The larger size of Na switches the (de)intercalation mechanism from a solid solution to a two‐phase transition. On charge, the disordered rocksalt structure of Na_2_MnO_2_F collapses, forming an amorphous material with significantly contracted Mn‐O/F bonds. Our results show that ion size fundamentally changes the redox chemistry and structural transitions that disordered rocksalts undergo, offering a route to control the electrochemical properties of these high‐energy‐density cathodes.

## 5 Experimental Section

### Synthesis

Na_2_MnO_2_F was prepared through mechanochemical ball milling. NaMnO_2_ and NaF (Aldrich, 99%) were combined in a 1:1 molar ratio and sealed in airtight zirconia jars (20 mL internal volume) within an argon‐filled glovebox, with H_2_O and O_2_ levels below 1 ppm. This mixture was then ball‐milled at 750 rpm for 6 h using a Fritsch Pulverisette 7 planetary ball mill, with 5 mm zirconia balls, using a powder‐to‐ball mass ratio of 1:20. The NaMnO_2_ precursor was synthesized by mixing anhydrous Na_2_CO_3_ (Fisher, 99.9%) and Mn_2_O_3_ (Aldrich, 99%) in a 1:1 molar ratio, followed by ball milling in an agate Retsch PM100 mill at 300 rpm for 4 h. Cold‐pressed pellets of the ground starting materials were then heated in an argon‐filled glovebox to 650 at 5 °C min^−1^, followed by a slower ramp to 800 at 1 °C min^−1^, with the temperature held at 800 °C for 10 h before naturally cooling back to room temperature.

Li_2_MnO_2_F was also synthesized by mechanochemical ball milling. Li_2_O (Fisher, 99.5%), Mn_2_O_3_ (Sigma Aldrich, 99%), and LiF (Sigma Aldrich, 99.99%) in a molar ratio of 1:1:2 was sealed in air‐tight zirconia jars in an argon‐filled glovebox with H_2_O and O_2_ less than 1 ppm. The mixture was then ball milled under the same conditions in the Fritsch Pulverisette 7 planetary ball mill as Na_2_MnO_2_F. All samples were stored in an argon‐filled glove box until further characterization.

### Electrochemistry

Self‐supporting electrode films were fabricated by mixing 70 wt.% active material, 20 wt.% carbon black as a conductive additive, and 10 wt.% polytetrafluoroethylene binder in a pestle and mortar. The electrode film was then calendered between rollers to a thickness of ≈0.15 mm. Electrochemical testing was performed in 2032 coin cells with glass microfiber separators (Whatman) soaked in electrolytes. For sodium cells, sodium metal discs (coated onto aluminum foil) were used as the negative electrode, with 1 m NaPF_6_ in a 1:1 mixture of ethylene carbonate: dimethyl carbonate (EC:DMC) by volume as the electrolyte (E‐lyte Innovations). For lithium cells, a lithium metal disc was used as the negative electrode, with 1 m LiPF_6_ in a 1:1 mixture of EC:DMC as the electrolyte (Aldrich). All electrode preparation was carried out in an argon‐filled glovebox. Galvanostatic charge–discharge measurements were performed at a rate of 10 mA g^−1^ (corresponding to C/22 for Li_2_MnO_2_F and C/18 for Na_2_MnO_2_F based on 1 mol of Na^+^/Li^+^) using a Maccor Series 4000 battery cycler at 30 °C. Cycling was carried out between 1.7 and 4.5 V versus Na^+^/Na or 2.0 and 4.8 V versus Li^+^/Li at a rate of 10 mA g^−1^ for all cells unless stated otherwise. Mass loadings were 5–10 mg cm^−2^.

### SEM‐EDX

Scanning electron microscopy (SEM) and energy‐dispersive X‐ray spectroscopy (EDX) analyses were conducted using a Carl Zeiss Merlin high‐resolution field emission microscope fitted with an Oxford Instruments X‐Max detector. SEM imaging and elemental mapping were performed with an accelerating voltage of 10–15 kV and a probe current of 100–400 pA for all samples.

### XAS and RIXS

X‐ray absorption spectra (XAS) were collected at the BL27SU beamline of the RIKEN/JASRI Spring‐8 synchrotron facility in Japan. Mn L‐edge data was collected in inverse partial fluorescence yield (IPFY) mode. Ex situ cathode samples were mounted on adhesive carbon tape and analyzed under ultra‐high vacuum (UHV) conditions. Resonant inelastic X‐ray scattering (RIXS) data were obtained at the I21 beamline, Diamond Light Source. Samples were transferred to the spectrometer using a vacuum transfer suitcase to avoid air exposure and were pumped down to UHV. RIXS line scans were collected at 530.75 eV at 16 different sample locations and averaged together. RIXS maps were collected at 0.1 eV intervals in excitation energy.

### X‐Ray Diffraction and PDF

Powder X‐ray diffraction (PXRD) patterns were collected using a Cu‐source Rigaku Miniflex diffractometer within a nitrogen‐filled glovebox. Rietveld profile refinements were performed using the general structure and analysis system (GSAS‐II) program.^[^ ^60^ ^]^ Operando PXRD measurements were conducted using a Cu‐source Rigaku 9 kW diffractometer with a beryllium window electrochemical cell provided by Rigaku. Ex situ PXRD data were acquired at the P02.1 beamline at Deutsches Elektronen‐Synchrotron (DESY). X‐ray total scattering data for pair distribution function (PDF) analysis were collected on the I15‐1 beamline at Diamond Lightsource. The ex situ PXRD and PDF samples were loaded into glass capillary tubes and sealed under an argon atmosphere. PDF data reduction was performed using PDFgetX3 program using a maximum momentum transfer of 20 A^−1^.^[^ ^61^ ^]^ Structural refinement was performed using the PDFgui program.^[^ ^62^ ^]^

### Iodometric Titration

Iodometric titration was used to determine the Mn valence in Na_2_MnO_2_F and Li_2_MnO_2_F. A known mass of each sample (≈5 mg) was dissolved in 25 mL of 1 m hydrochloric acid (degassed with argon). After dissolution, a few drops of starch indicator and an excess of potassium iodide were added to the solution. During the titration, the mixture was stirred and continuously purged with argon, while 0.02 m sodium thiosulfate was added dropwise until the solution became colorless. The concentration of the sodium thiosulfate solution was standardized using potassium iodate prior to the titration.

## Conflict of Interest

The authors declare no conflict of interest.

## Supporting information



Supporting Information

## Data Availability

The data that support the findings of this study are available from the corresponding author upon reasonable request.
