# Supplementary material for: Influence of Ion Size on Structure and Redox Chemistry in Na‐Rich and Li‐Rich Disordered Rocksalt Battery Cathodes
Source: Adv Mater. 2025 May 30;37(32):2419878. doi: 10.1002/adma.202419878 (PMC12355563; doi:10.1002/adma.202419878)
Supplement: Supplementary file 1 — Supporting Information [file ADMA-37-2419878-s001.docx]

**Supplementary Information**

**Influence of ion size on structure and redox chemistry in Na-rich and Li-rich disordered rocksalt battery cathodes**

Nicole C. Mitchell^1,2^, Oliver O. Thomas^1,2^, Benjamin G. Meyer^1,2^, Mirian Garcia-Fernandez^3^, Ke-Jin Zhou^3^, Patrick S. Grant^1,2^, Peter G. Bruce^1,2^, Richard Heap^4^, Ruth Sayers^4^, Robert A. House^1,2^ *

^1^Department of Materials, University of Oxford, Parks Road, Oxford, OX1 3PH, UK

^2^The Faraday Institution, Didcot, OX11 0RA, UK

^3^Diamond Light Source, Harwell Campus, Didcot, OX11 0DE, UK

^4^Faradion Ltd., The Innovation Centre, Sheffield, S1 4DP, UK

*Corresponding Author [robert.house@materials.ox.ac.uk](mailto:robert.house@materials.ox.ac.uk)


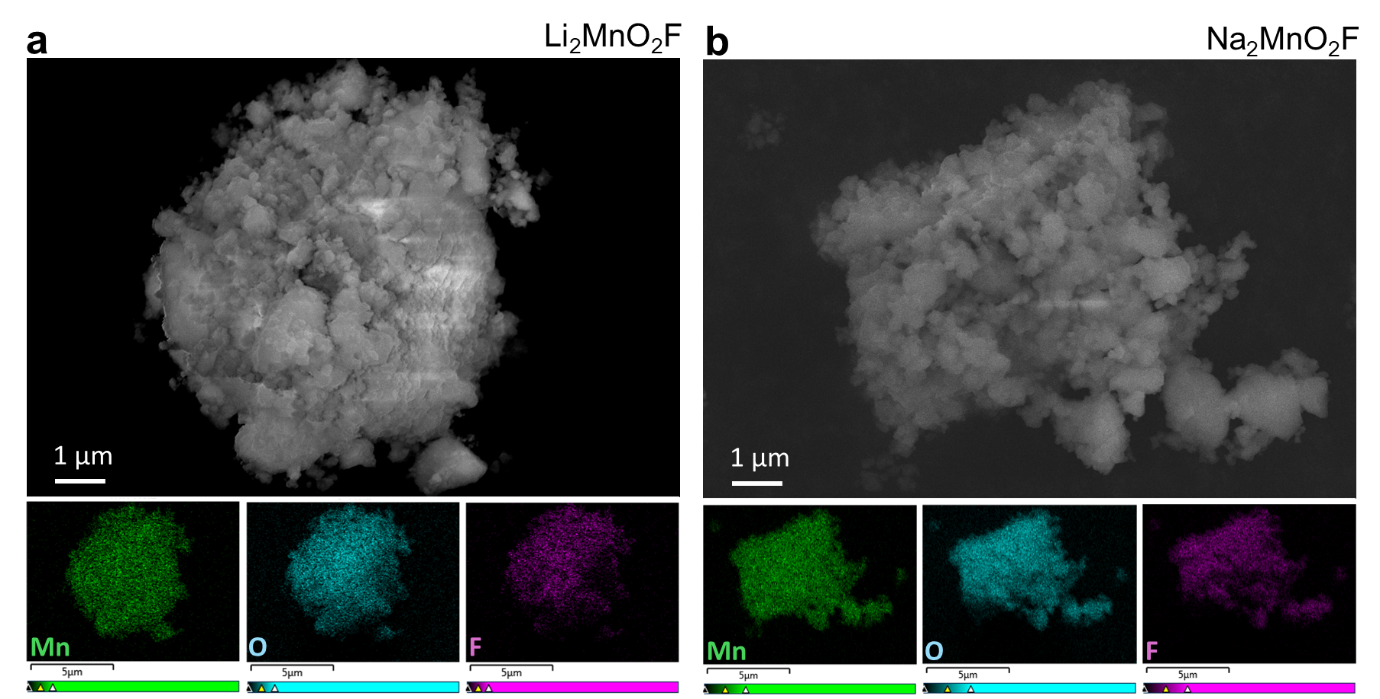


Figure S1. Energy dispersive X-ray spectroscopy analysis of pristine (a) Li_2_MnO_2_F, (b) Na_2_MnO_2_F.

Table S1. Rietveld refinement parameters for XRD data of pristine (a) Li_2_MnO_2_F, (b) Na_2_MnO_2_F.

| **Atom** | **Wyckoff Position** | **x** | **y** | **z** | **Occupancy** | **U_iso_** **(Å^2^)** |
| --- | --- | --- | --- | --- | --- | --- |
| Li | 4a | 0 | 0 | 0 | 0.6667 | 0.0177(1) |
| Mn | 4a | 0 | 0 | 0 | 0.3333 | 0.00825(2) |
| O | 4b | 0.5 | 0.5 | 0.5 | 0.6667 | 0.0480(6) |
| F | 4b | 0.5 | 0.5 | 0.5 | 0.3333 | 0.0004(7) |
| a = 4.15193(2) Å, Space Group Fm-3m | | | | | | |
| R_w_ = 2.329 %, χ^2^ = 0.87 | | | | | | |

**a**

| **Atom** | **Wyckoff Position** | **x** | **y** | **z** | **Occupancy** | **U_iso_** **(Å^2^)** |
| --- | --- | --- | --- | --- | --- | --- |
| Na | 4a | 0 | 0 | 0 | 0.6667 | 0.0079(3) |
| Mn | 4a | 0 | 0 | 0 | 0.3333 | 0.01201(3) |
| O | 4b | 0.5 | 0.5 | 0.5 | 0.6667 | 0.0292(8) |
| F | 4b | 0.5 | 0.5 | 0.5 | 0.3333 | 0.0896(2) |
| a = 4.50001(3) Å, Space Group Fm-3m | | | | | | |
| R_w_ = 4.040 %, χ^2^ = 0.62 | | | | | | |

**b**


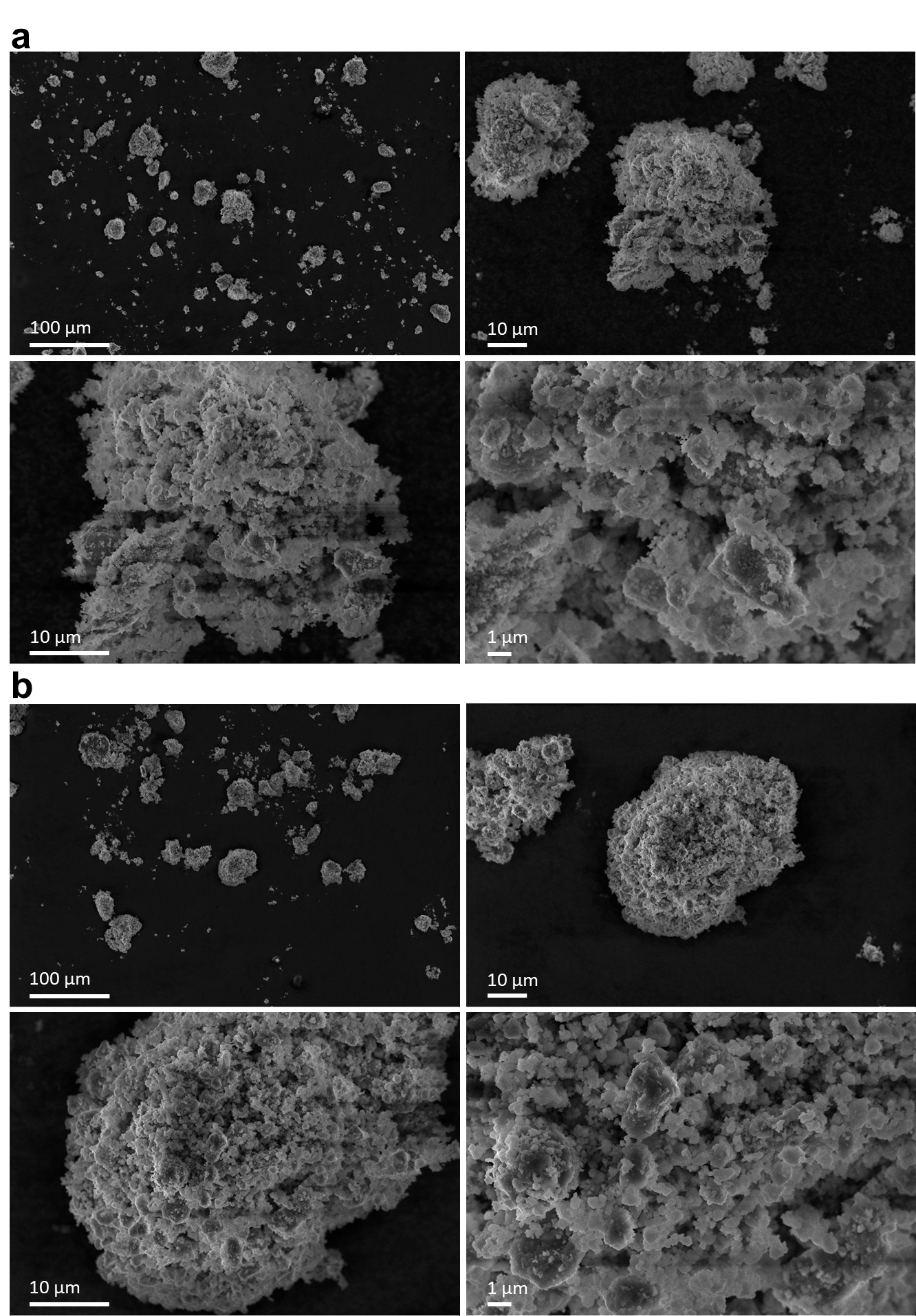


Figure S2. Scanning electron microscopy (SEM) images of pristine (a) Li_2_MnO_2_F, (b) Na_2_MnO_2_F.

Table S2. Oxidation states for Mn measured using iodometric titration. Three repeat measurements and an average are given.

| **Na_2_MnO_2_F** | **Li_2_MnO_2_F** |
| --- | --- |
| 3.013 | 3.022 |
| 3.023 | 2.997 |
| 2.970 | 2.990 |
| 3.00(2) | 3.00(1) |





Figure S3. Charge-discharge profiles Li_2_MnO_2_F and Na_2_MnO_2_F with 1 mol extraction. The Na_2_MnO_2_F curve is offset by 0.3 V to account for the Na vs. Li voltage difference.


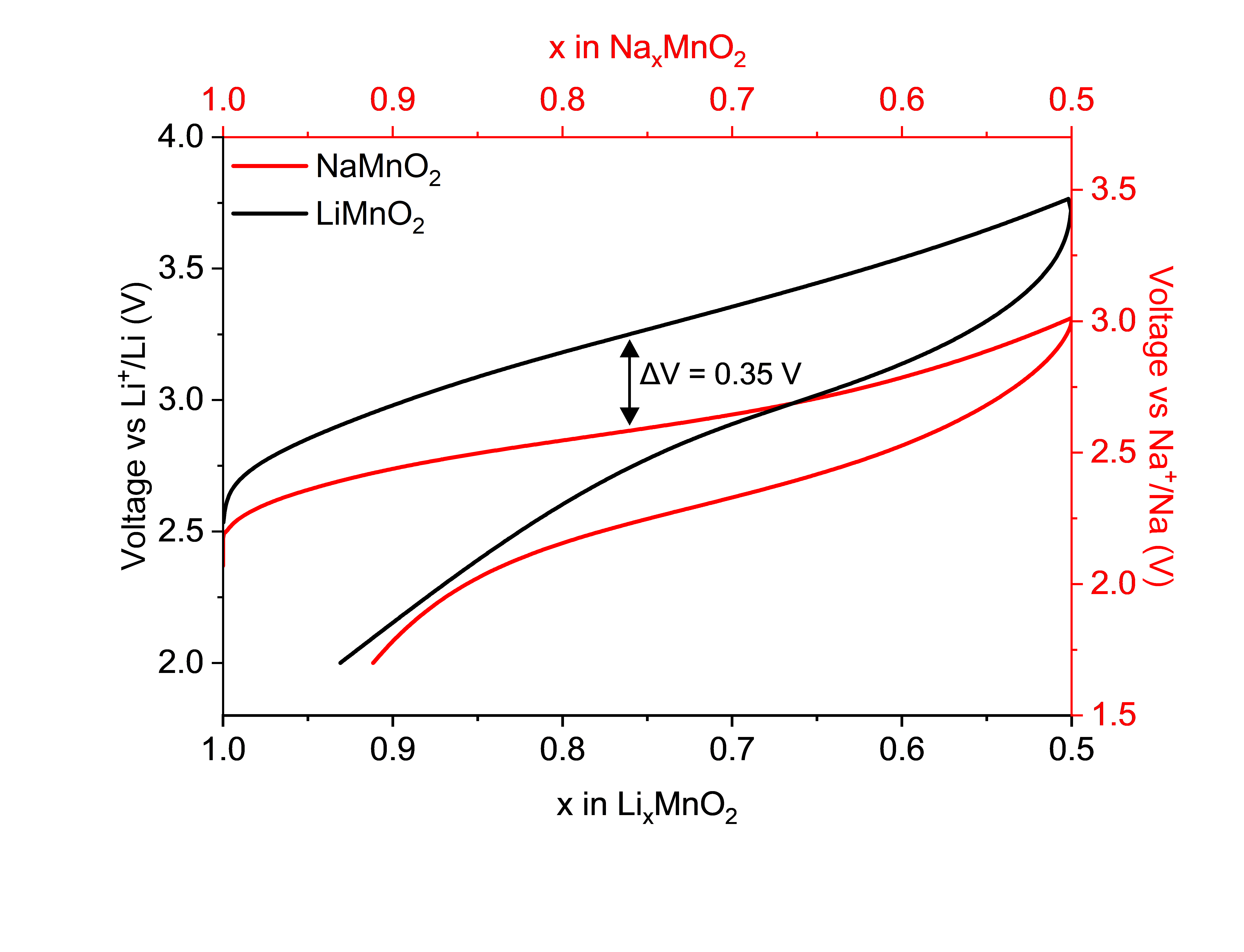



Figure S4. First cycle load curves for LiMnO_2_ and NaMnO_2_ with average charge voltages of 3.25 V and 2.6 V respectively, a difference of 0.35 V correcting for the anode potential.

Figure S5. RIXS spectra collected at 530.75 eV for Na_2_MnO_2_F and Li_2_MnO_2_F at the end of the nominal Mn-redox capacity when 1 Na/Li has been removed.

Table S3. X-ray PDF refinement parameters for pristine Li_2_MnO_2_F.

| **Atom** | **Wyckoff Position** | **x** | | **y** | **z** | **Occupancy** | **U_iso_** **(Å^2^)** |
| --- | --- | --- | --- | --- | --- | --- | --- |
| Li | 4a | 0 | 0 | | 0 | 0.6667 | 0.02(1) |
| Mn | 4a | 0 | 0 | | 0 | 0.3333 | 0.02(2) |
| O | 4b | 0.5 | 0.5 | | 0.5 | 0.6667 | 0.08(4) |
| F | 4b | 0.5 | 0.5 | | 0.5 | 0.3333 | 0.019(9) |
| a = 4.145(4) Å, Space Group = Fm-3m, sp diameter = 42(3) Å, scale factor = 0.28(4) | | | | | | | |
| R_w_ = 0.27 | | | | | | |  |
| Q_max_ = 20 Å^-1^ | | | | | | |  |
| R = 1.7 – 30 Å | | | | | | |  |

Table S4. X-ray PDF refinement parameters for Li_2_MnO_2_F charged to 4.8 V.

| **Atom** | **Wyckoff Position** | **x** | **y** | **z** | **Occupancy** | **U_iso_** **(Å^2^)** |
| --- | --- | --- | --- | --- | --- | --- |
| Li | 4a | 0 | 0 | 0 | 0.6667 | 0.051(7) |
| Mn | 4a | 0 | 0 | 0 | 0.3333 | 0.052(7) |
| O | 4b | 0.5 | 0.5 | 0.5 | 0.6667 | 0.016(3) |
| F | 4b | 0.5 | 0.5 | 0.5 | 0.3333 | 0.016(3) |
| a = 4.041(4) Å, Space Group = Fm-3m, sp diameter = 50(5) Å, scale factor = 0.20(1) | | | | | | |
| **Atom** | **Wyckoff Position** | **x** | **y** | **z** | **Occupancy** | **U_iso_** **(Å^2^)** |
| Li | 4d | 0.65(5) | 0.41(2) | 0.25 | 0.26 | 0.01(2) |
| Mn | 4d | 0.65(5) | 0.41(2) | 0.25 | 0.4 | 0.05(2) |
| Li | 8e | 0.21(3) | 0.147(8) | 0.068(8) | 0.26 | 0.01(2) |
| Mn | 8e | 0.21(3) | 0.147(8) | 0.068(8) | 0.4 | 0.05(2) |
| O | 4c | 0.72(4) | 0.25 | 0 | 1 | 0.006(4) |
| O | 4d | 0.24(4) | 0.203(8) | 0.25 | 1 | 0.006(4) |
| O | 8e | 0.24(2) | 0.512(4) | 0.073(3) | 1 | 0.006(4) |
| a = 2.80(3) Å, b = 9.6(1) Å, c= 9.9(1) Å, Space Group = Pbcm, sp diameter = 10.8(9) Å, scale factor = 0.90(7) | | | | | | |
| R_w_ = 0.17  Q_max_ = 20 Å^-1^  R = 1.7 – 30 Å | | | | | | |

Table S5. X-ray PDF refinement parameters for pristine Na_2_MnO_2_F.

| **Atom** | **Wyckoff Position** | **x** | **y** | **z** | **Occupancy** | **U_iso_** **(Å^2^)** |
| --- | --- | --- | --- | --- | --- | --- |
| Na | 4a | 0 | 0 | 0 | 0.6667 | 0.031(7) |
| Mn | 4a | 0 | 0 | 0 | 0.3333 | 0.032(6) |
| O | 4b | 0.5 | 0.5 | 0.5 | 0.6667 | 0.10(3) |
| F | 4b | 0.5 | 0.5 | 0.5 | 0.3333 | 0.10(5) |
| a = 4.486(8) Å, Space Group = Fm-3m, sp diameter = 37(3) Å, scale factor = 0.28(2) | | | | | | |
| R_w_ = 0.23 | | | | | | |
| Q_max_ = 20 Å^-1^ | | | | | | |
| R = 1.7 – 30 Å | | | | | | |

Table S6. X-ray PDF refinement parameters for Na_2_MnO_2_F charged to 4.5 V.

| **Atom** | **Wyckoff Position** | **x** | **y** | **z** | **Occupancy** | **U_iso_** **(Å^2^)** |
| --- | --- | --- | --- | --- | --- | --- |
| Mn | 4d | 0.62(5) | 0.40(3) | 0.25 | 0.4(2) | 0.02(3) |
| Mn | 8e | 0.22(5) | 0.13(1) | 0.06(1) | 0.4(2) | 0.02(3) |
| O | 4c | 0.69(4) | 0.25 | 0 | 1 | 0.003(6) |
| O | 4d | 0.22(5) | 0.21(1) | 0.25 | 1 | 0.003(6) |
| O | 8e | 0.26(3) | 0.506(6) | 0.073(5) | 1 | 0.003(6) |
| a = 2.81(5) Å, b = 9.4(2) Å, c= 10.0(2) Å, Space Group = Pbcm, sp diameter = 12(3) Å, scale factor = 0.6(2) | | | | | | |
| R_w_ = 0.19  Q_max_ = 20 Å^-1^  R = 1.7 – 30 Å | | | | | | |





Figure S6. Fitted X-ray PDF data of Li_2_MnO_2_F charged to 3.7 V.

Table S7. X-ray PDF refinement parameters for Li_2_MnO_2_F charged to 3.7 V.

| **Atom** | **Wyckoff Position** | **x** | **y** | **z** | **Occupancy** | **U_iso_** **(Å^2^)** |
| --- | --- | --- | --- | --- | --- | --- |
| Li | 4a | 0 | 0 | 0 | 0.6667 | 0.04(4) |
| Mn | 4a | 0 | 0 | 0 | 0.3333 | 0.044(7) |
| O | 4b | 0.5 | 0.5 | 0.5 | 0.6667 | 0.015(3) |
| F | 4b | 0.5 | 0.5 | 0.5 | 0.3333 | 0.016(3) |
| a = 4.111(6) Å, Space Group = Fm-3m, sp diameter = 50(9) Å, scale factor = 0.15(3) | | | | | | |
| **Atom** | **Wyckoff Position** | **x** | **y** | **z** | **Occupancy** | **U_iso_** **(Å^2^)** |
| Li | 4d | 0.70(5) | 0.41(2) | 0.25 | 0.6 | 0.03(2) |
| Mn | 4d | 0.70(5) | 0.41(2) | 0.25 | 0.4 | 0.03(2) |
| Li | 8e | 0.22(3) | 0.149(8) | 0.068(8) | 0.6 | 0.03(2) |
| Mn | 8e | 0.22(3) | 0.149(8) | 0.068(8) | 0.4 | 0.03(2) |
| O | 4c | 0.72(4) | 0.25 | 0 | 1 | 0.007(4) |
| O | 4d | 0.16(4) | 0.211(8) | 0.25 | 1 | 0.007(4) |
| O | 8e | 0.24(2) | 0.511(4) | 0.081(3) | 1 | 0.007(4) |
| a = 2.77(3) Å, b = 9.7(1) Å, c = 10.0(1) Å, Space Group = Pbcm, sp diameter = 7.8(6) Å, scale factor = 1.5(2) | | | | | | |
| R_w_ = 0.24  Q_max_ = 20 Å^-1^  R = 1.7 – 30 Å | | | | | | |



Figure S7. Fitted X-ray PDF data of Li_2_MnO_2_F charged to 4.5 V.

Table S8. X-ray PDF refinement parameters for Li_2_MnO_2_F charged to 4.5 V.

| **Atom** | **Wyckoff Position** | **x** | **y** | **z** | **Occupancy** | **U_iso_** **(Å^2^)** |
| --- | --- | --- | --- | --- | --- | --- |
| Li | 4a | 0 | 0 | 0 | 0.6667 | 0.07(5) |
| Mn | 4a | 0 | 0 | 0 | 0.3333 | 0.043(7) |
| O | 4b | 0.5 | 0.5 | 0.5 | 0.6667 | 0.016(3) |
| F | 4b | 0.5 | 0.5 | 0.5 | 0.3333 | 0.015(3) |
| a = 4.055(4) Å, Space Group = Fm-3m, sp diameter = 52(9) Å, scale factor = 0.16(4) | | | | | | |
| **Atom** | **Wyckoff Position** | **x** | **y** | **z** | **Occupancy** | **U_iso_** **(Å^2^)** |
| Li | 4d | 0.68(5) | 0.41(2) | 0.25 | 0.4 | 0.03(2) |
| Mn | 4d | 0.68(5) | 0.41(2) | 0.25 | 0.4 | 0.02(2) |
| Li | 8e | 0.22(3) | 0.149(8) | 0.068(8) | 0.4 | 0.03(2) |
| Mn | 8e | 0.22(3) | 0.149(8) | 0.068(8) | 0.4 | 0.02(2) |
| O | 4c | 0.72(4) | 0.25 | 0 | 1 | 0.009(4) |
| O | 4d | 0.19(4) | 0.204(8) | 0.25 | 1 | 0.009(4) |
| O | 8e | 0.24(2) | 0.509(4) | 0.08(3) | 1 | 0.009(4) |
| a = 2.80(3) Å, b = 9.6(1) Å, c = 9.9(1) Å, Space Group = Pbcm, sp diameter = 7.7(5) Å, scale factor = 1.4(1) | | | | | | |
| R_w_ = 0.21  Q_max_ = 20 Å^-1^  R = 1.7 – 30 Å | | | | | | |



Figure S8. Fitted X-ray PDF data of Na_2_MnO_2_F charged to 3 V.

Table S9. X-ray PDF refinement parameters for Na_2_MnO_2_F charged to 3 V.

| **Atom** | **Wyckoff Position** | **x** | **y** | **z** | **Occupancy** | **U_iso_** **(Å^2^)** |
| --- | --- | --- | --- | --- | --- | --- |
| Na | 4a | 0 | 0 | 0 | 0.6667 | 0.03(2) |
| Mn | 4a | 0 | 0 | 0 | 0.3333 | 0.09(3) |
| O | 4b | 0.5 | 0.5 | 0.5 | 0.6667 | 0.10(5) |
| F | 4b | 0.5 | 0.5 | 0.5 | 0.3333 | 0.1(1) |
| a = 4.47(2) Å, Space Group = Fm-3m, sp diameter = 35(6) Å, scale factor = 0.20(3) | | | | | | |
| **Atom** | **Wyckoff Position** | **x** | **y** | **z** | **Occupancy** | **U_iso_** **(Å^2^)** |
| Na | 4d | 0.73(5) | 0.42(3) | 0.25 | 0.6 | 0.10(3) |
| Mn | 4d | 0.73(5) | 0.42(3) | 0.25 | 0.4 | 0.03(3) |
| Na | 8e | 0.23(5) | 0.14(1) | 0.04(1) | 0.6 | 0.10(3) |
| Mn | 8e | 0.23(5) | 0.14(1) | 0.04(1) | 0.4 | 0.03(3) |
| O | 4c | 0.73(4) | 0.25 | 0 | 1 | 0.001(6) |
| O | 4d | 0.07(5) | 0.20(1) | 0.25 | 1 | 0.001(6) |
| O | 8e | 0.25(3) | 0.493(6) | 0.072(5) | 1 | 0.001(6) |
| a = 2.76(5) Å, b = 10.6(2) Å, c = 10.7(2) Å, Space Group = Pbcm, sp diameter = 6.7(6) Å, scale factor = 1.6(3) | | | | | | |
| R_w_ = 0.21  Q_max_ = 20 Å^-1^  R = 1.7 – 30 Å | | | | | | |





Figure S9. Fitted X-ray PDF data of Na_2_MnO_2_F charged to 3.8 V.

Table S10. X-ray PDF refinement parameters for Na_2_MnO_2_F charged to 3.8 V.

| **Atom** | **Wyckoff Position** | **x** | **y** | **z** | **Occupancy** | **U_iso_** **(Å^2^)** |
| --- | --- | --- | --- | --- | --- | --- |
| Na | 4d | 0.67(5) | 0.44(1) | 0.25 | 0.4 | 0.10(3) |
| Mn | 4d | 0.67(5) | 0.44(1) | 0.25 | 0.4 | 0.01(2) |
| Na | 8e | 0.26(2) | 0.144(4) | 0.039(5) | 0.4 | 0.10(3) |
| Mn | 8e | 0.26(2) | 0.144(4) | 0.039(5) | 0.4 | 0.01(2) |
| O | 4c | 0.76(3) | 0.25 | 0 | 1 | 0.004(9) |
| O | 4d | 0.19(3) | 0.21(2) | 0.25 | 1 | 0.004(9) |
| O | 8e | 0.22(3) | 0.497(4) | 0.081(6) | 1 | 0.004(9) |
| a = 2.83 Å, b = 9.8(2) Å, c= 11.0(2) Å, Space Group = Pbcm, sp diameter = 6(1) Å, scale factor = 3.5(4) | | | | | | |
| R_w_ = 0.21  Q_max_ = 20 Å^-1^  R = 1.7 – 30 Å | | | | | | |



Figure S10. Fitted X-ray PDF data of Na_2_MnO_2_F charged to 4.25 V.

Table S11. X-ray PDF refinement parameters for Na_2_MnO_2_F charged to 4.25 V.

| **Atom** | **Wyckoff Position** | **x** | **y** | **z** | **Occupancy** | **U_iso_** **(Å^2^)** |
| --- | --- | --- | --- | --- | --- | --- |
| Na | 4d | 0.63(4) | 0.40(2) | 0.25 | 0.2 | 0.02(3) |
| Mn | 4d | 0.63(4) | 0.40(2) | 0.25 | 0.4 | 0.02(3) |
| Na | 8e | 0.21(5) | 0.135(8) | 0.057(6) | 0.2 | 0.02(3) |
| Mn | 8e | 0.21(5) | 0.135(8) | 0.057(6) | 0.4 | 0.02(3) |
| O | 4c | 0.69(4) | 0.25 | 0 | 1 | 0.003(6) |
| O | 4d | 0.22(6) | 0.21(1) | 0.25 | 1 | 0.003(6) |
| O | 8e | 0.26(3) | 0.505(5) | 0.075(5) | 1 | 0.003(6) |
| a = 2.82(5) Å, b = 9.4(2) Å, c= 9.9(2) Å, Space Group = Pbcm, sp diameter = 11(2) Å, scale factor = 0.7(3) | | | | | | |
| R_w_ = 0.21  Q_max_ = 20 Å^-1^  R = 1.7 – 30 Å | | | | | | |





Figure S11. Fitted X-ray PDF data of Na_2_MnO_2_F charged to 4.5 V and then discharged to 1.7 V.

Table S12. X-ray PDF refinement parameters for Na_2_MnO_2_F charged 4.5 V and then discharged to 1.7 V.

| **Atom** | **Wyckoff Position** | **x** | **y** | **z** | **Occupancy** | **U_iso_** **(Å^2^)** |
| --- | --- | --- | --- | --- | --- | --- |
| Na | 4a | 0 | 0 | 0 | 0.6667 | 0.06(3) |
| Mn | 4a | 0 | 0 | 0 | 0.3333 | 0.06(3) |
| O | 4b | 0.5 | 0.5 | 0.5 | 0.6667 | 0.11(7) |
| F | 4b | 0.5 | 0.5 | 0.5 | 0.3333 | 0.1(1) |
| a = 4.47(3) Å, Space Group = Fm-3m, sp diameter = 26(5) Å, scale factor = 0.20(4) | | | | | | |
| **Atom** | **Wyckoff Position** | **x** | **y** | **z** | **Occupancy** | **U_iso_** **(Å^2^)** |
| Na | 4d | 0.62(5) | 0.47(3) | 0.25 | 0.6 | 0.002(7) |
| Mn | 4d | 0.62(5) | 0.47(3) | 0.25 | 0.4 | 0.002(5) |
| Na | 8e | 0.26(5) | 0.14(1) | 0.03(1) | 0.6 | 0.002(7) |
| Mn | 8e | 0.26(5) | 0.14(1) | 0.03(1) | 0.4 | 0.002(5) |
| O | 4c | 0.75(4) | 0.25 | 0 | 1 | 0.002(6) |
| O | 4d | 0.25(5) | 0.24(1) | 0.25 | 1 | 0.002(6) |
| O | 8e | 0.20(3) | 0.496(6) | 0.061(5) | 1 | 0.002(6) |
| a = 2.96(3) Å, b = 10.1(2) Å, c= 11.2(2) Å, Space Group = Pbcm, sp diameter = 6(1) Å, scale factor = 0.9(3) | | | | | | |
| R_w_ = 0.21  Q_max_ = 20 Å^-1^  R = 1.7 – 30 Å | | | | | | |


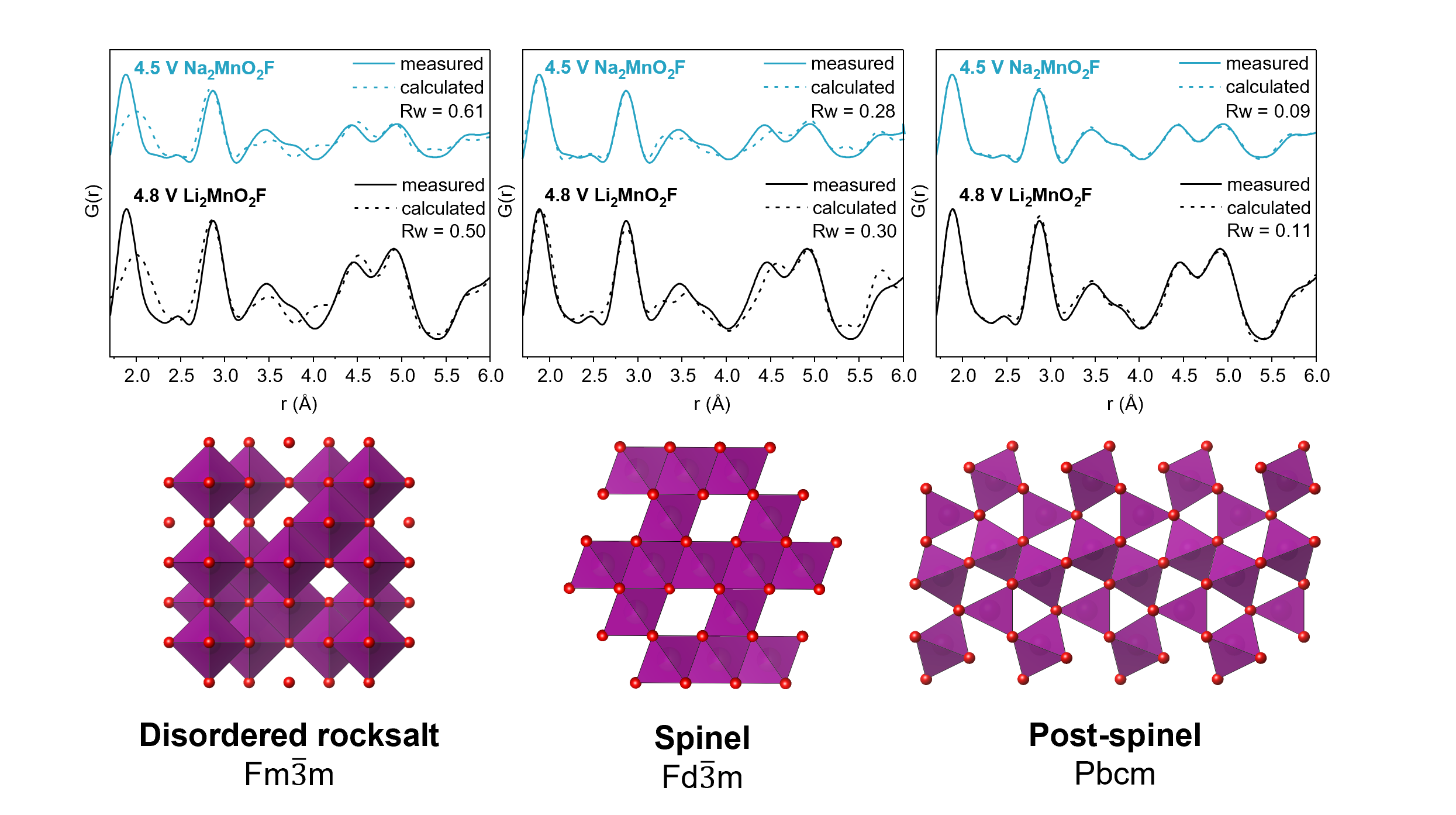


Figure S12. X-ray PDF fits for Na_2_MnO_2_F charged to 4.5 V and Li_2_MnO_2_F charged to 4.8 V using a disordered rocksalt (Fm3m), spinel (Fd3m) and post-spinel (pbcm) structure.

Figure S13. Fittings of the local structure of the X-ray PDF for pristine Li_2_MnO_2_F and Na_2_MnO_2_F.

Table S13. Measured average heights and volumes of the tetrahedral interstices in Na_2_MnO_2_F and Li_2_MnO_2_F from X-ray diffraction and the ion size of Na and Li.

|  | **Tetrahedral height ( Å )** | **Tetrahedral volume ( Å ^3^)** | **Ionic Radius ( Å )** |
| --- | --- | --- | --- |
| **Na_2_MnO_2_F** | 2.60 | 3.8 | 1.02 |
| **Li_2_MnO_2_F** | 2.38 | 2.9 | 0.76 |
| **Ratio** | 1.1 | 1.3 | 1.3 |


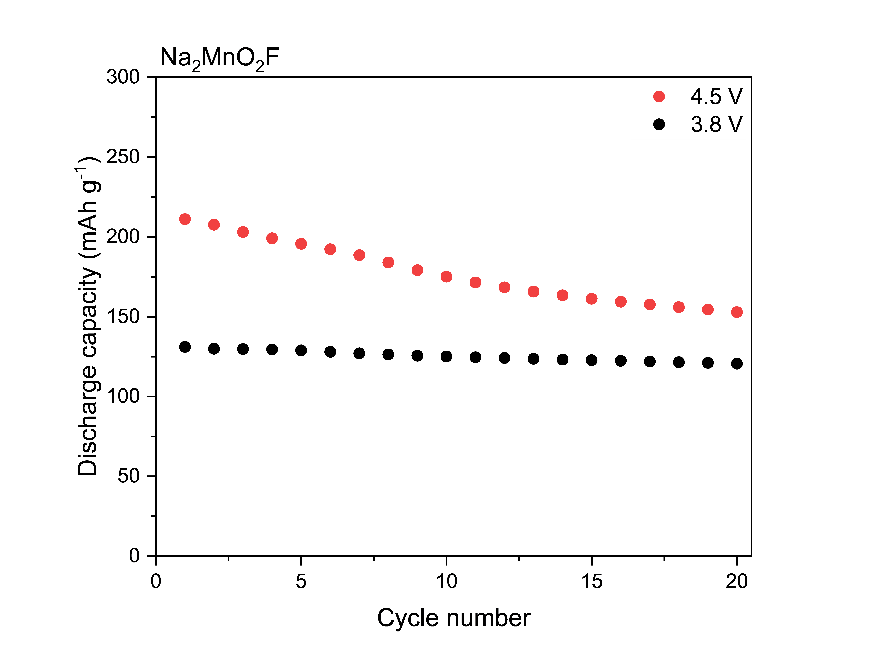

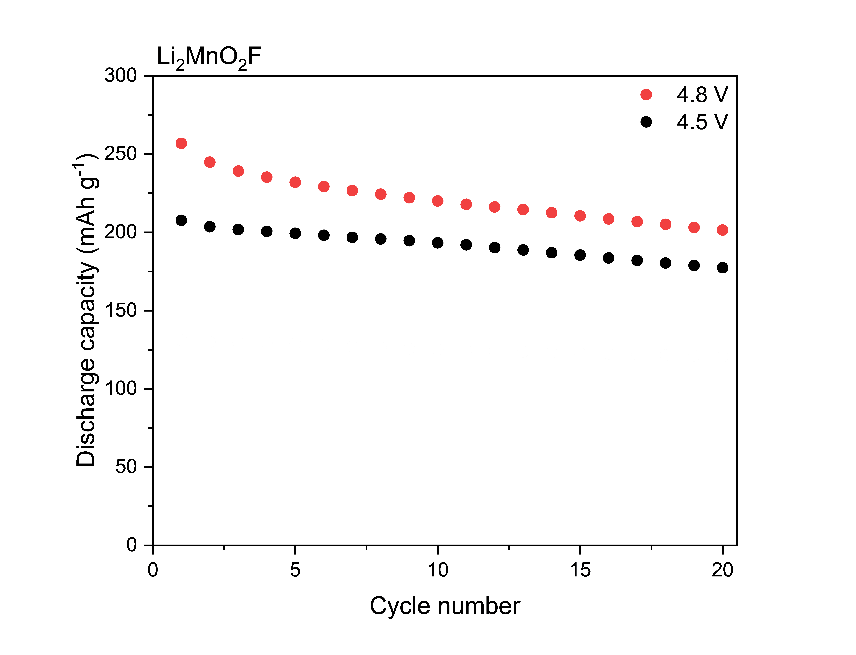


Figure S14. Capacity retention for Na_2_MnO_2_F and Li_2_MnO_2_F cycled to different upper cutoff voltages.


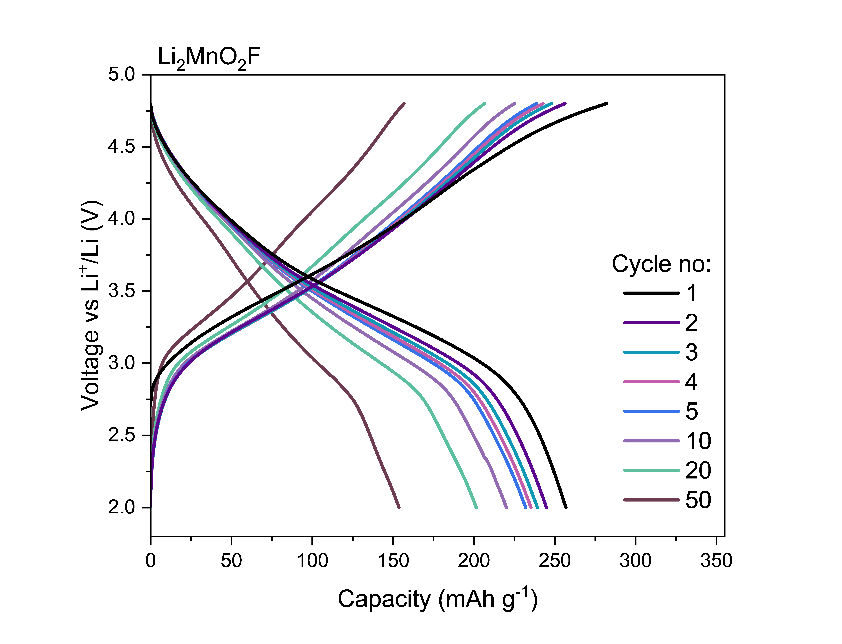

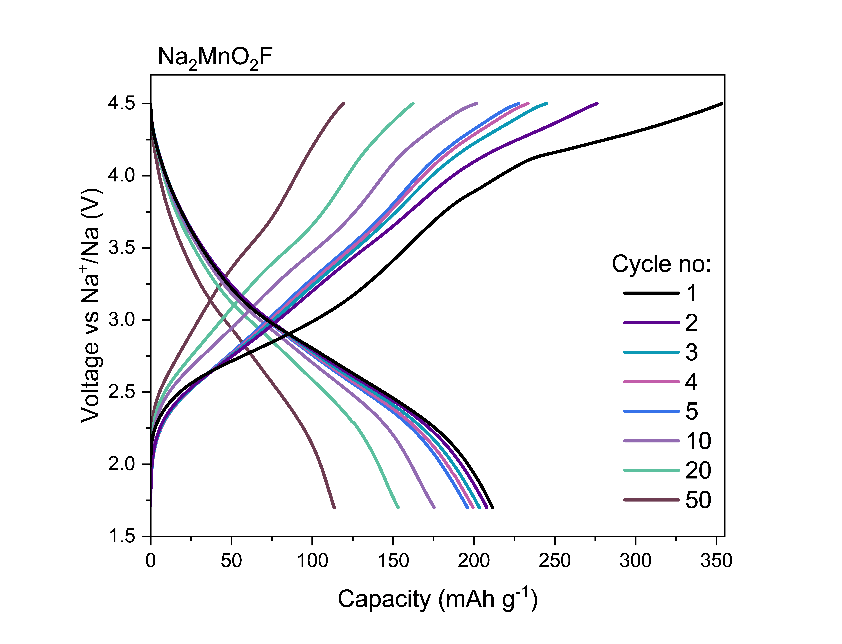


Figure S15. Load curves for Na_2_MnO_2_F and Li_2_MnO_2_F over the first 50 cycles.


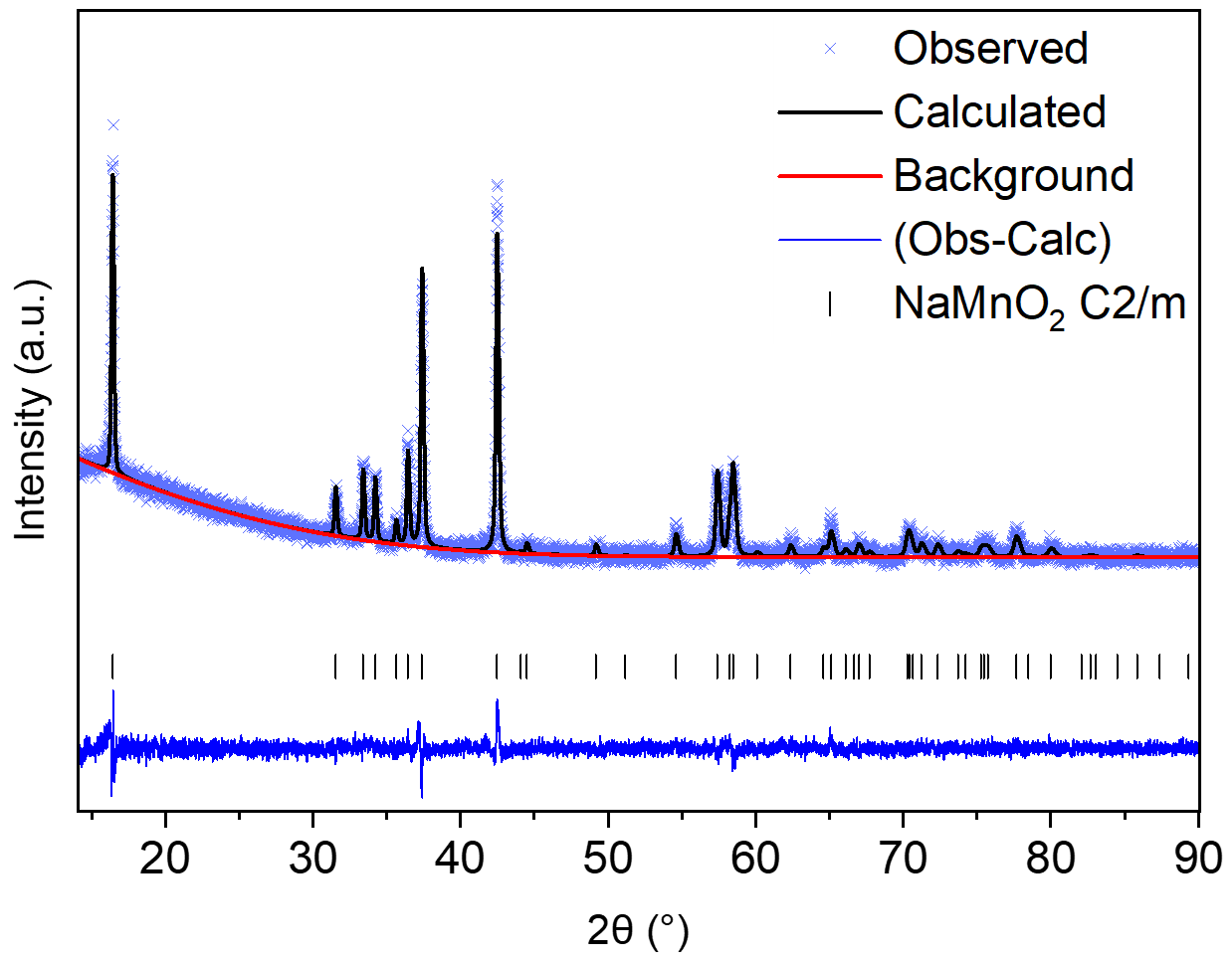


Figure S16. Fitted XRD data of NaMnO_2_ precursor.

Table S14. Rietveld refinement parameters for XRD data of NaMnO_2_.

| **Atom** | **Wyckoff Position** | **x** | **y** | **z** | **Occupancy** | **U_iso_** **(Å^2^)** |
| --- | --- | --- | --- | --- | --- | --- |
| Mn | 2a | 0 | 0 | 0 | 1 | 0.0074(1) |
| Na | 4d | 0 | 0.5 | 0.5 | 1 | 0.0024(2) |
| O | 4i | 0.28647 | 0 | 0.79049 | 1 | 0.0182(2) |
| a = 5.6677(2) Å, b = 2.85630(1) Å, c = 5.8007(2) Å, β = 113.167, Space Group C 2/m | | | | | | |
| R_w_ = 5.736 %, χ^2^ = 1.06 | | | | | | |
